# Enhanced Wound Healing- and Inflammasome-Associated Gene Expression in TNFAIP3-Interacting Protein 1- (TNIP1-) Deficient HaCaT Keratinocytes Parallels Reduced Reepithelialization

**DOI:** 10.1155/2020/5919150

**Published:** 2020-04-21

**Authors:** Rambon Shamilov, Tyler W. Ackley, Brian J. Aneskievich

**Affiliations:** ^1^Graduate Program in Pharmacology & Toxicology, School of Pharmacy, University of Connecticut, Storrs, CT 06269, USA; ^2^Doctor of Pharmacy Program, School of Pharmacy, University of Connecticut, Storrs, CT 06269, USA; ^3^Department of Pharmaceutical Sciences, School of Pharmacy, University of Connecticut, Storrs, CT 06269, USA

## Abstract

TNIP1 protein is a widely expressed, cytoplasmic inhibitor of inflammatory signaling initiated by membrane receptors such as TLRs which recognize pathogen-associated and damage-associated molecular patterns (PAMPs and DAMPs). Keratinocyte TNIP1 deficiency sensitizes cells to PAMPs and DAMPs promoting hyperresponsive expression and secretion of cytokine markers (e.g., IL-8 and IL-6) relevant to cases of chronic inflammation, like psoriasis, where TNIP1 deficiency has been reported. Here, we examined the impact of TNIP1 deficiency on gene expression and cellular responses (migration and viability) relevant to acute inflammation as typically occurs in wound healing. Using siRNA-mediated TNIP1 expression knockdown in cultured HaCaT keratinocytes, we investigated TNIP1 deficiency effects on signaling downstream of TLR3 agonism with low-concentration poly (I:C), a representative PAMP/DAMP. The combination of TNIP1 knockdown and PAMP/DAMP signaling disrupted expression of specific keratinocyte differentiation markers (e.g., transglutaminase 1 and involucrin). These same conditions promoted synergistically increased expression of wound-associated markers (e.g., S100A8, TGF*β*, and CCN2) suggesting potential benefit of increased inflammatory response from reduced TNIP1 protein. Unexpectedly, poly (I:C) challenge of TNIP1-deficient cells restricted reepithelialization and reduced cell viability. In these cells, there was not only increased expression for genes associated with inflammasome assembly (e.g., ASC, procaspase 1) but also for A20, a TNIP1 partner protein that represses cell-death signaling. Despite this possibly compensatory increase in A20 mRNA, there was a decrease in phospho-A20 protein, the form necessary for quenching inflammation. Hyperresponsiveness to poly (I:C) in TNIP1-deficient keratinocytes was in part mediated through p38 and JNK pathways. Taken together, we conclude that TNIP1 deficiency promotes enhanced expression of factors associated with promoting wound healing. However, the coupled, increased potential priming of the inflammasome and reduced compensatory activity of A20 has a net negative effect on overall cell recovery potential manifested by poor reepithelialization and viability. These findings suggest a previously unrecognized role for TNIP1 protein in limiting inflammation during successful progression through early wound healing stages.

## 1. Introduction

Epidermal keratinocytes are constantly exposed to pattern recognition molecules from local microbes, debris released from minor injuries, or self-generated factors from organelle breakdown during terminal differentiation (cornification) [1]. Separately and together, these damage-associated molecular patterns (DAMPs) and pathogen-associated molecular patterns (PAMPs) have the potential to trigger an innate immune response and a devastating, potentially cell-lethal, inflammatory cascade [1, 2]. Thus, epidermal keratinocytes must be competent to limit effects of these low-level instigators to maintain or, in the event of large scale trauma or infection, restore an immunoquiescent state.

Tumor necrosis factor *α*-induced protein 3-interacting protein 1 (TNIP1) is a negative modulator of cytoplasmic inflammatory signaling downstream of several cell membrane receptors. TNIP1, alternatively named Naf, VAN, and ABIN-1, [3–5] is expressed in multiple epithelial and nonepithelial tissues as well as cultured cells derived from them [6, 7]. TNIP1 limits cytoplasmic signal progression downstream of TLR, TNF-R, and EGF-R, thus protecting cells from inopportune NF-*κ*B-, IRF3-, and ERK-mediated transcription (see [4, 8] for review). Expression studies in multiple cell and tissue types have demonstrated that experimentally derived TNIP1 deficiency or knockout initiates and/or enhances inflammatory phenotypes [9–13], often recapitulating pathologies known for their relapsing or chronic immune activation. Given that human pathologies such as psoriasis, lupus, and scleroderma [12, 14, 15] are associated with reduced TNIP1 protein levels, as opposed to being expression null, we modeled cells deficient in TNIP1 protein and their responses to limiting amounts of likely encountered cell membrane receptor signaling molecules to assess for possible synergistic effects of the two conditions. We previously reported [13] that numerous inflammatory chemokines, cytokines, and their related receptors were preferentially expressed in TNIP1-deficient HaCaT keratinocytes challenged with low amounts of TLR agonists, the bacterial lipoprotein FSL-1 or the synthetic double-stranded RNA poly (I:C) (polyinosinic-polycytidylic acid), as compared to control conditions and those cells with either only TNIP1 knockdown or TLR agonist exposure. This suggests that epidermal keratinocytes might be particularly sensitive to TNIP1 deficiency when encountering PAMPs and DAMPs associated with topical microbes or experiencing tissue-damaging trauma.

Here, we asked if TNIP1 deficiency in HaCaT keratinocytes would impact gene expression and reepithelialization associated with wound healing, which includes transient and often overlapping inflammation and proliferation phases. We challenged control and TNIP1-deficient HaCaT keratinocytes with 1 *μ*g/mL poly (I:C), a concentration which alone promotes TLR3 activity, but does not induce the maximal inflammatory signaling response seen with dual TNIP1 deficiency and poly (I:C) exposure [13]. This TLR3 agonist models microbial PAMPs due to local infection as well as cellular DAMPs released from injury associated with wound initiation [16, 17]. TNIP1 deficiency reduced the expression of a few keratinocyte-specific markers. In contrast, there were significant and often many-fold increases in transcripts associated with cell damage signaling, antimicrobial responses, epithelial-to-mesenchymal transition, inflammasome components, and wound healing in general. At the cellular level, these responses led to reduced viability and reduced reepithelization. We conclude that experimental TNIP1 deficiency, paralleling that in diverse pathologies, hypersensitizes cells to otherwise subthreshold levels of DAMPs/PAMPs, exacerbating intracellular inflammatory signaling to the detriment of wound recovery. In turn, this may initiate or intensify gene expression consequences that could limit tolerance to encounters with cutaneous microbes, slow recovery from minor damage events, and/or contribute to chronic wound or fibrotic states.

## 2. Materials and Methods

### 2.1. Cell Culture and Treatments

HaCaT keratinocytes, a spontaneously immortalized, nontumorigenic line [18], were cultured as previously described [13]. The HaCaT keratinocyte line has been extensively used to model human keratinocyte inflammatory pathway signaling, cytokine production, and reepithelialization [19–26]. TNIP1-targeting siRNA or nontargeting negative control siRNA was used to transfect HaCaT keratinocytes (triplicate wells plated as follows: 6-well, 480,000 cells/well; 12-well, 160,000 cells/well; 24-well, 80,000 cells/well) 24 hr postplating. SMARTpool TNIP1 siRNA (100 nM final concentration) or nontargeting siRNA (GE Dharmacon, Lafayette, CO) in Opti-MEM serum-free media (Thermo Fisher Scientific, Waltham, MA) was used for transfection with DharmaFECT2 reagent. TLR3 ligand poly (I:C) at 1 *μ*g/mL (or nuclease-free H_2_O as vehicle control) was added to the cells in serum-free medium 48 hr post-siRNA transfection, which included a 24 hr serum-free rest period immediately prior to addition of agonist. For poly (I:C) exposure duration, see figure legends. For inhibitor studies, cells were incubated with either MAPK p38 inhibitor SB203580 at 10 *μ*M (InvivoGen, San Diego, CA), JNK inhibitor SP600125 at 30 *μ*M (InvivoGen, San Diego, CA), or DMSO (vehicle control, final concentration 0.05%) for 2 hr immediately prior to addition of poly (I:C). Cells were collected at 0, 6, 12, or 24 hr in RIPA lysis buffer (10 mM Tris, 150 mM NaCl, 1% deoxycholic acid, 1% Triton, and 0.1% sodium dodecyl sulfate) with Roche cOmplete Protease Inhibitor Cocktail (Roche Life Science, Indianapolis, IN) and protein phosphatase inhibitors (2 mM sodium orthovanadate and 50 mM sodium fluoride) or RNA RLT lysis buffer (QIAGEN, Hilden, Germany) for protein and RNA isolation, respectively.

### 2.2. Quantitative Real-Time Polymerase Chain Reaction (qRT-PCR)

RNA was extracted using RNeasy (QIAGEN), with cells lysed directly in RLT buffer for either immediate use or storage at -20°C. Isolated RNA was quantified using a NanoDrop Microvolume Spectrophotometer (Thermo Fisher Scientific). cDNA was generated using a High-Capacity cDNA Kit (Applied Biosystems, Foster City, CA), diluted from 20 *μ*L to a final volume of 120 *μ*L. Gene amplification was performed using the Applied Biosystems 7500 Fast Real-Time PCR System with 0.4 *μ*M of each forward and reverse primer (Supplementary Table 1) and 2 *μ*L of diluted cDNA. Ribosomal protein L13a (RPL13a) was used for normalization during analysis by the delta-delta Ct method. Cycling conditions were as follows: 95°C for 20 seconds and (95°C for 3 seconds, 60°C for 30 seconds) × 40 cycles. Ct threshold was set at Ct ≤ 36 cycles.

### 2.3. qRT-PCR Microarray

For gene expression analysis with the Human Wound Healing PCR array (QIAGEN, cat no: PAHS-121Z), cDNA was generated using the RT2 First Strand Kit (QIAGEN) from RNA samples of TNIP1 and nontargeting siRNA-transfected cells collected 6 hr post-poly (I:C) treatment as described in Cell Culture and Treatments. SABiosciences PCR Array software was used to analyze gene expression data by the delta-delta Ct method after normalization with RPLP0. Ct cutoff was set at Ct ≤ 35.

### 2.4. Western Blots

Protein concentration from RIPA buffer lysate supernatants was determined using the Pierce Modified Lowry Assay (Thermo Fisher Scientific). Clarified supernatants were combined with 5x Laemmli sample buffer (0.3125 M Tris-HCl pH 6.8, 10% SDS, 50% glycerol, 0.005% bromophenol blue, and 10% *β*-mercaptoethanol) to a final 1x for immediate use or storage at -80°C. After resolution of 16.5 *μ*g of protein per lane on 10% polyacrylamide gels, proteins were transferred onto a Whatman Protran nitrocellulose membrane (GE Healthcare Life Sciences, Marlborough, MA). Membranes were blocked with 5% nonfat milk (or 5% BSA when detecting phosphorylated protein) solution in Tris-buffered saline with 0.05% Tween-20 (TBS-T) for 1 hr prior to addition and incubation with a primary antibody overnight at 4°C. Antibodies used include *β*-actin at 1 : 5000 dilution (Cell Signaling Technology, Danvers, MA, cat no: 4967S), A20/TNFAIP3 at 1 : 1000 dilution (Cell Signaling Technology, cat no: 5630S), Phospho-A20 (Ser381) at 1 : 1000 dilution (Cell Signaling Technology, cat no: 63523S), and TNIP1 at 1 : 1000 dilution [6]. After washing with TBS-T, blots were incubated with HRP-conjugated secondary antibody at 1 : 5000 dilution (Thermo Fisher Scientific, cat no: 31460) for 2 hr at room temperature. Proteins were visualized using chemiluminescence substrates (Thermo Fisher Scientific) on a Kodak IS400CF CCD imager (Kodak, Rochester, NY). Densitometry assessment was performed using Carestream Molecular Imaging Software. Background subtraction for all bands was done on individual background areas prior to normalization with *β*-actin.

### 2.5. Scratch Assay and Confluence Measurements

HaCaT keratinocytes were set in triplicate for each condition in 12-well plates, transfected as above (Cell Culture and Treatments), maintained for 24 hr in standard media, and then switched to serum-free media for another 24 hr during which time they reached confluence. A “wound” was created in HaCaT monolayers [25, 26] by dragging the point of a sterile yellow micropipette tip across the well center. Immediately after performing the scratch, cells were washed twice with PBS before addition (time 0) of serum-free media with poly (I:C) at 1 *μ*g/mL or nuclease-free H_2_O as vehicle control for 24 hr. Reepithelialization for each condition was monitored with 15 images along the scratch length, in each of the triplicates, taken with a 10x objective on a Nikon Eclipse TS100 Microscope with SPOT 4.5.9.12 software and a SPOT Insight digital camera. Analysis of the remaining “wound” area was done using TScratch software [27]. Analysis of cell confluence was performed using PHANTAST software [28].

### 2.6. Cell Viability Assay (MTS)

HaCaT keratinocytes were plated in 96-well plates at ~40-50% confluence. TNIP1 deficiency was induced as in Cell Culture and Treatments. Cell viability was assessed using the CellTiter 96 AQueous One Solution Cell Proliferation Assay (MTS) (Promega, Madison, WI) as per the manufacturer's instructions. Absorbance (490 nm) was measured on a SpectraMax 190 Microplate Reader (Molecular Devices, San Jose, CA).

### 2.7. ELISA

Cell culture media were collected and clarified with centrifugation at 300 × g at 4°C; samples were immediately aliquoted and stored at -80°C. CCN2 ELISA was performed using the ABTS ELISA Buffer Kit (PeproTech, Rocky Hill, NJ) and Human CTGF (i.e., CCN2) Mini ABTS ELISA Development Kit (PeproTech, cat no: 900-M317), as per the manufacturer's instructions.

### 2.8. Statistical Analysis

Statistical analysis was performed using GraphPad Prism (version 7) with results presented as mean with standard error of the mean (SEM). Student's *t*-test or two-way ANOVA followed by Bonferroni's or Tukey's post hoc analysis was used when performing specific pairwise comparisons or comparisons of all conditions, respectively. Outcomes were described as previously suggested [29, 30]. A *p* value ≤ 0.05 was considered as statistically significant.

## 3. Results

### 3.1. TNIP1 Deficiency Impacts Poly (I:C)-Induced Gene Expression Changes Associated with Keratinocyte Differentiation and Activation

Disease-associated and experimentally generated changes in TNIP1 protein levels have indicated its repression of chronic inflammatory signaling in epidermis and other tissues (see [4, 8, 12, 31] for review). TNIP1-deficient epidermal keratinocytes are hyperresponsive to TLR agonists in their production of chemokines and cytokines [13, 32]. Increased cytokine expression is typically associated with the activated keratinocyte state [33–35] seen in transient and persistent inflammation associated with successful healing and protracted wounds, respectively. The activated keratinocyte is one triggered, by physical trauma or by soluble factors, to undergo numerous gene expression changes leading to alteration in cell-specific proteins (e.g., keratins) as well as the production of a wide range of cytokines/chemokines involved in the local spread of inflammatory signals which may in turn impact local cell replication, migration, extracellular matrix remodeling, and in tissues, immune cell recruitment [36–38].

To probe TNIP1 function and how it might relate to acute inflammation and activated keratinocytes, we generated an experimental model of its deficiency by TNIP1-targeting siRNA transfection of HaCaT keratinocytes and examined a range of cellular consequences in response to TLR agonism (Supplementary Figure (1a)). As compared to control cells receiving nontargeting siRNA, TNIP1 protein was reduced by 75% and 70% at 48 hr and 72 hr posttransfection, respectively (Supplementary Figures 1(b) and (c)), in the TNIP1 siRNA sets.

**Figure 1:**
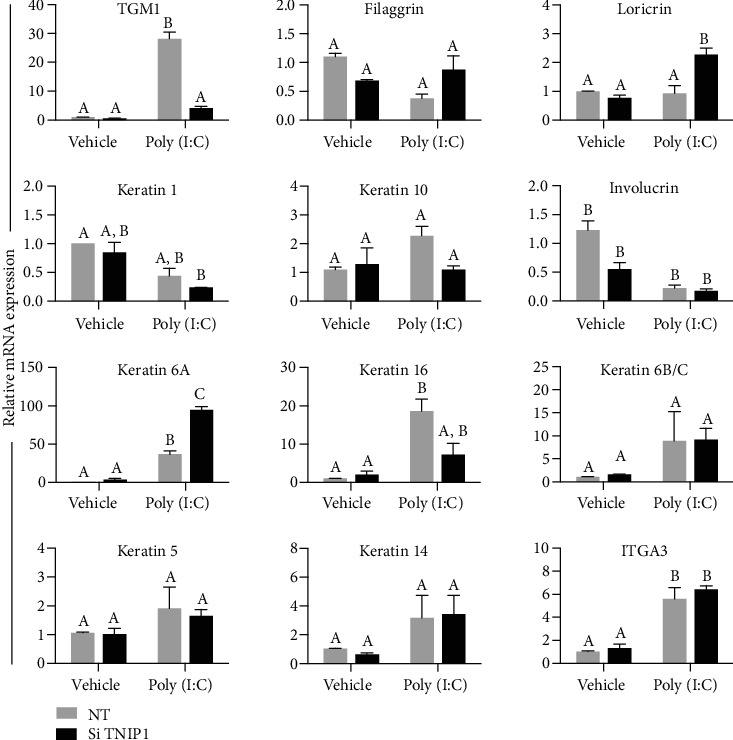
Keratinocyte differentiation marker gene expression response to TNIP1 deficiency and poly (I:C). Gene expression was measured from HaCaT keratinocyte RNA collected 12 hr post-poly (I:C) (1 *μ*g/mL) treatment (see Materials and Methods). mRNA levels were measured by qRT-PCR, normalized to RPL13a, and shown as fold change relative to nontargeting, vehicle. Data is presented as mean + SEM from two independent experiments. Means with a common letter are not significantly different by two-way ANOVA followed by Tukey's post hoc test at a significance level of *p* ≤ 0.05. TGM1: transglutaminase 1; ITGA3: integrin *α*3. Transfections were done with nontargeting (NT) or TNIP1 small interfering RNA (Si TNIP1).

To better define the state of TNIP1-deficient HaCaT keratinocytes compared to nontargeting siRNA-transfected cells under vehicle and TLR agonist-challenged conditions, we determined the relative levels of transcripts associated with characteristic stages of keratinocyte step-wise maturation. Control and TNIP1-deficient HaCaT keratinocytes were exposed to the DAMP/PAMP poly (I:C), a TLR3 ligand and dsRNA mimic. Using qPCR analysis, we determined that with TNIP1 deficiency, HaCaT keratinocyte expression of basal layer markers keratin 5 and 14 (K5 and K14, respectively) appears unaffected under vehicle or poly (I:C) conditions (Figure 1, bottom row). In the case of ITGA3, the *α*3 partner of the integrin receptor for the basal lamina protein laminin, there was a significant ~6-fold mRNA increase from poly (I:C) exposure which was matched in nontargeting and TNIP1-deficient cells.

K6 and K16 are associated with keratinocyte activation by affecting migration and being respondent to and possibly participating in inflammatory response [39]. We found that poly (I:C) significantly stimulated gene expression of K6A and K16 in control siRNA keratinocytes. TNIP1 deficiency further enhanced this poly (I:C) effect for K6A but not for K16 or K6B/C. Of the paired K1 and K10 suprabasal markers, K1 in TNIP1-deficient poly (I:C) cells was reduced compared to nontargeting siRNA-transfected cells under vehicle conditions; K10 expression was unaffected. Involucrin transcripts significantly decreased ~2-fold from TNIP1 deficiency alone, with a trend for further decrease in the poly (I:C)-treated, TNIP1-deficient cells. The differentiation marker transglutaminase-1 (TGM1) transcript level was dramatically increased by ~20-fold with poly (I:C) alone while TNIP1 deficiency greatly restricted that agonist-induced response. Late-stage differentiation markers filaggrin and loricrin remained mostly unchanged from either poly (I:C) exposure or TNIP1 knockdown with the one exception of ~2-fold increased loricrin transcripts upon dual TNIP1 deficiency and poly (I:C) stimulation. These latter two markers, while reliably detected within the qRT-PCR parameters used (see Materials and Methods), likely represent a small cohort of late differentiation-competent cells relative to more abundant middifferentiation stage cells indicated by the abundance of transcripts for earlier stage markers such as suprabasal K1 and K10 (Supplementary Figure 2). Thus, while expression of some characteristic keratinocyte markers was affected by either TNIP1 deficiency alone or TNIP1 deficiency affecting response to poly (I:C) challenge, there was no unidirectional change that would be indicative of either blanket enhancement or limit to maturation across the cell population.

### 3.2. TNIP1 Deficiency Promotes Poly (I:C)-Induced Gene Expression Associated with Diverse Functionality Related to Wound Healing

**Figure 2:**
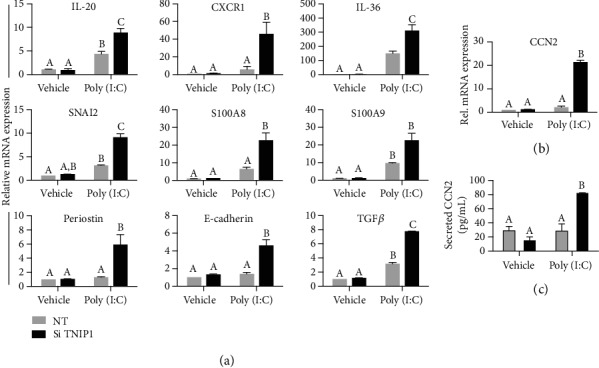
TNIP1 deficiency and low-level exposure to poly (I:C) promotes increased expression of EMT, epithelial tissue remodeling, and inflammation markers. (a, b) Gene expression was measured from HaCaT keratinocyte RNA collected 6 hr post-poly (I:C) (1 *μ*g/mL) treatment. mRNA levels were measured by qRT-PCR, normalized to RPL13a, and shown as fold change relative to nontargeting siRNA and vehicle-treated cells. (c) Secreted CCN2 was detected using supernatant collected 12 hr post-poly (I:C) (1 *μ*g/mL) treatment from cells transfected with either nontargeting or TNIP1 siRNA. Data is presented as mean + SEM from two independent experiments with conditions in each set in triplicate. For (a), means with a common letter are not significantly different by two-way ANOVA followed by Tukey's post hoc test at a significance level of *p* ≤ 0.05. For (b) and (c), *p* ≤ 0.01.

Keratinocyte contribution to epidermal recovery postwounding is a synergy of cell-specific differentiation proteins, as well as a capacity to upregulate expression of antimicrobial peptides, inflammatory cytokines, and tissue injury and repair factors. Here, we found that TNIP1 deficiency promoted significantly increased gene expression associated with these often overlapping states. Periostin and E-cadherin gene expression which often changes in wound healing remained unaffected by either TNIP1 deficiency or poly (I:C) exposure alone (Figure 2(a)); however, for these combined conditions, there were significant increases in periostin and E-cadherin expression, 6- and 4-fold, respectively. To investigate what other changes TNIP1 deficiency might promote, we assessed expression of TGF*β* and EMT-promoting SNAI2 (i.e., SLUG) [40] and found for each significant increases above already poly (I:C)-induced significant expression under dual TNIP1 deficiency and TLR3 agonism (Figure 2(a)). A similar expression profile was observed with antimicrobial genes S100A8 and A9, where poly (I:C) stimulation of TNIP1-deficient keratinocytes promoted a 4- and 2-fold increase, respectively, as compared to the poly (I:C) alone treated cells. IL-20, associated with controlling HaCaT keratinocyte proliferation [41], showed significant increases due to poly (I:C) stimulation with further enhancement with TNIP1 deficiency. CXCR1 (i.e., IL-8 receptor) was measured at 40-fold greater gene expression with TNIP1 deficiency and poly (I:C), relative to untreated keratinocytes, with an ~8-fold increase as compared to poly (I:C) alone. IL-36*γ*, a potent inducer of keratinocyte activation [42], was doubled with the combination of poly (I:C) and TNIP1 deficiency, compared to already significant changes from poly (I:C)-treated cells only. The profibrotic factor CCN2 is significantly upregulated in keratinocytes participating in wound healing [43]. Low constitutive expression of CCN2 at the mRNA and protein levels in control HaCaT cells (nontargeting siRNA; vehicle) was unaffected by separate TNIP1 knockdown or exposure to low-concentration poly (I:C). However, there was an ~10-fold increase in CCN2 mRNA and an ~4-fold increase in secreted CCN2 protein for cells dually experiencing TNIP1 protein deficiency and challenge with low-dose poly (I:C) (Figures 2(b) and 2(c)). Taken together, these transcripts follow a general trend of enhanced expression of genes associated with wound healing (proliferative, migratory, and antimicrobial markers) in poly (I:C) stimulated, TNIP1-deficient cells suggesting wide-ranging consequences beyond cell differentiation and inflammation.

### 3.3. TNIP1 Deficiency during Poly (I:C) Exposure Promotes Differential Expression of Wound Healing-Associated Genes and Limits In Vitro Reepithelialization

Wound healing in epithelia consists of progressive and usually overlapping phases of inflammation, proliferation/migration, ECM deposition, and tissue remodeling [44] throughout which there would be characteristic gene expression changes. Differences in wound healing-associated gene expression were assessed via RT-qPCR, comparing TNIP1-deficient cells versus those with endogenous TNIP1 levels in their response to poly (I:C) (Table 1). TNIP1-deficient keratinocytes challenged with poly (I:C) displayed increased expression of genes for proinflammatory responses (e.g., TNF*α*, CXCL11, and IL-6) as well as increases for the anti-inflammatory cytokine IL-10 [45] and the growth factor TGF*α*. Induction of the latter is seen in activated keratinocytes due to diverse stimuli [46]. Among the keratinocyte-specific integrins tested, only integrin *α*5 reached the standard ≥2-fold cutoff for an increase. However, the *α*5 partner *β*1 and *α*3, another *β*1 partner, were increased ~50 and 60%, respectively (see Supplementary Figure 3 for the full array of assessed genes).

Strikingly, several transcripts whose proteins are positively or negatively involved with ECM remodeling (Table 1) were increased in poly (I:C)-challenged, TNIP1-deficient HaCaT keratinocytes. For instance, SERPINE1 (~9.4-fold increase) encodes plasminogen activator inhibitor 1 (PAI-1), the major inhibitor of urokinase plasminogen activator (PLAU), which increased ~2-fold. Additionally, PLAUR, the receptor for PLAU, increased ~3.3-fold. F3, coagulation factor III (alias tissue factor), increased ~2.5-fold; it participates in signal transduction outside of blood coagulation such as inflammation and cell migration [47]. Not all ECM-remodeling enzymes on the array were universally increased in response to poly (I:C) challenge of TNIP1-deficient HaCaT keratinocytes. Matrix metallopeptidase (MMP) 1, MMP2, and MMP9 had similar expression among the two groups (Supplementary Figure 3). Transcripts for ECM-related enzymes MMP7, cathepsin (CTSK), and coagulation factor XIII A subunit (F13A1) decreased ~4-fold or more for poly (I:C)-challenged, TNIP1-deficient HaCaT keratinocytes which also shared a 2-3-fold decrease in the promigration factors CXCL2, CXCL5, and CCL2. Thus, as with other instigators of keratinocyte activation such as EGF [46], there is a coinciding induction of genes in poly (I:C)-exposed, TNIP1-deficient HaCaT keratinocytes that either facilitate or limit wound healing.

**Figure 3:**
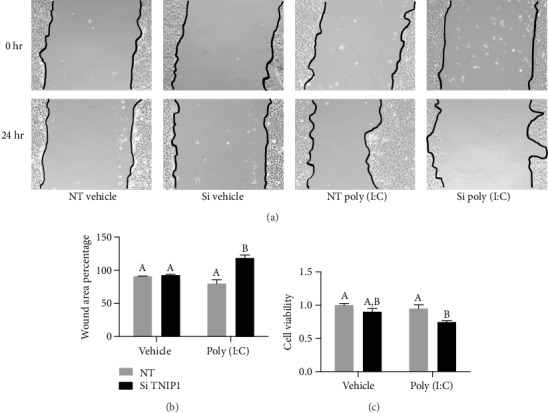
TNIP1 deficiency significantly inhibits HaCaT keratinocyte reepithelialization postscratch in the presence of low levels of TLR3 agonist poly (I:C). (a) A scratch “wound” in HaCaT keratinocyte monolayers was generated two days after nontargeting (NT) or TNIP1 (Si) siRNA transfection and then exposed to either vehicle or poly (I:C) (1 *μ*g/mL). Representative images (*n* = 45, 15 from each triplicate well) were taken at this time (0 hr) and again one day (24 hr) later. (b) Wound area percentage remaining (0 hr considered 100%) 24 hr postscratch was calculated using TScratch software. (c) Cell viability was determined by MTS assay 72 hr posttransfection of HaCaT cells with either nontargeting or TNIP1 siRNA, exposed to poly (I:C) (1 *μ*g/mL) (or vehicle control) for the last 24 hr. Data is presented as mean + SEM from two independent experiments with conditions in each set in triplicate. Statistical analysis was performed using two-way ANOVA followed by Bonferroni's post hoc test. Asterisk (∗) represents statistically significant difference (^∗^*p* value of ≤0.05; ^∗∗^*p* value of ≤0.005; NS: nonsignificant).

To test TNIP1 impact during reepithelialization, we used an in vitro wound model [25, 26] with confluent HaCaT keratinocytes under conditions of normal (nontargeting siRNA) or deficient TNIP1 (TNIP1 siRNA) protein levels (Figures 3(a) and 3(b)). Cells with reduced TNIP1 protein under vehicle conditions had scratch area recovery similar to those with normal levels of TNIP1 protein. Cells in this latter case tended to be better at reepithelialization in the presence of poly (I:C). In contrast, TNIP1-deficient HaCaT cells in the presence of poly (I:C) displayed not only reduced refill of the scratch area but further widening of the denuded area (increased wound area percentage). For vehicle conditions (i.e., no poly (I:C)), cell density behind the scratch was confluent right up to the wound edge for the nontargeting and TNIP1 siRNA groups. However, paralleling expansion of the wound area seen with TNIP1 deficiency and poly (I:C) exposure, there was a significant decrease in cell confluence (Supplementary Figures 4(a) and 4(b)) relative to nontargeting siRNA and poly (I:C) at 24 hr. In separate experiments of intact HaCaT monolayers (nonwounded) post-TNIP1 knockdown and poly (I:C) exposure, we assessed culture viability. Within the poly (I:C) sets, TNIP1 deficiency promoted a significant decrease in viability (Figure 3(c)). These scratch and viability results suggest that TNIP1-deficient keratinocytes would have poor wound healing performance in damaged epithelia despite the increase in some gene expression products which positively contribute to tissue recovery.

### 3.4. TNIP1 Deficiency Promotes Increased Expression of Propyroptotic Gene Transcripts

**Figure 4:**
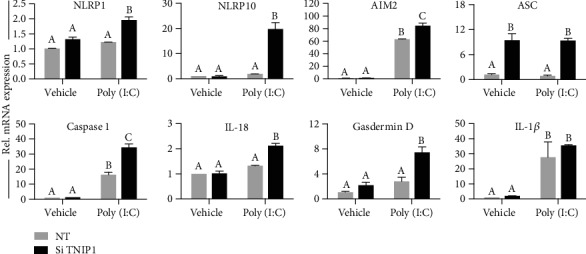
Upregulated gene expression of pyroptosis-associated markers with TNIP1 deficiency and poly (I:C) treatment. Gene expression changes were measured from RNA isolated 6 hr post-poly (I:C) (1 *μ*g/mL) treatment of TNIP1-deficient HaCaT keratinocytes. mRNA levels were measured by qRT-PCR, normalized to RPL13a, and shown as fold change relative to siRNA negative control and vehicle-treated cells (NT). Data is presented as mean + SEM from two independent experiments. Means with a common letter are not significantly different by two-way ANOVA followed by Tukey's post hoc test at a significance level of *p* ≤ 0.05.

Expression of inflammasome components, key to mediating a proinflammatory form of programmed cell death [48] known as pyroptosis, has been reported downstream of keratinocyte exposure to high poly (I:C) concentrations [49]. We tested the possibility of a similar response but with the low poly (I:C) concentration used here where its effects seemed magnified by TNIP1 deficiency. TNIP1-deficient, poly (I:C)-treated HaCaT keratinocytes had significantly increased transcripts for nucleotide-binding oligomerization domain-like receptor proteins (NLRP) NLRP1 and 10 (Figure 4). In contrast, the low-concentration poly (I:C) exposure alone did not promote increased transcription of these two gene targets, as compared to the vehicle control cells. Transcripts for potential inflammasome components, NLRC4 and NLRP3, the latter commonly associated with dsRNA sensing, were not detected (not shown). However, expression of the functionally related absent in melanoma 2 (AIM2) was significantly increased in response to poly (I:C) and further significantly increased for this condition by TNIP1 deficiency. Interestingly, for apoptosis-associated speck-like protein containing a CARD domain (ASC), an adaptor protein to NLR proteins, gene expression was maximized to similar levels (~8.5-fold increase) by TNIP1 deficiency alone irrespective of poly (I:C) exposure. Expression of caspase-1, needed for enzymatic processing within the inflammasome, was induced with poly (I:C) treatment of TNIP1 control keratinocytes with the induction being ~100% higher in the TNIP1-deficient cells. Expression of caspase-1 substrates IL-18 and gasdermin D, but not IL-1*β*, shared an increase in expression due to TNIP1 deficiency in poly (I:C)-treated cells. Thus, as with inflammation and several other cell damage responses or wound healing target gene responses (Figure 2 and Table 1), there is potentiation in TNIP1-deficient cells of several propyroptosis genes.

### 3.5. Decrease in Phosphorylated A20 Occurs with TNIP1 Deficiency in Poly (I:C)-Challenged Cells

**Figure 5:**
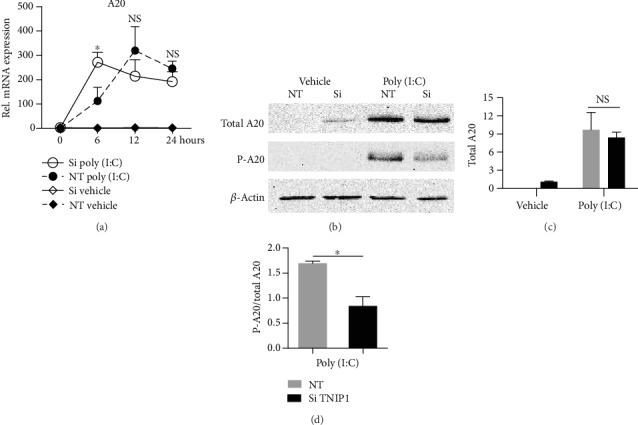
A20 phosphorylation is decreased in cells with reduced TNIP1. (a) Gene expression analysis at 6, 12, and 24 hours post-poly (I:C) (1 *μ*g/mL) exposure of HaCaT keratinocytes transfected with TNIP1 siRNA (Si) or nontargeting control (NT). qRT-PCR was analyzed by the delta-delta Ct method after normalization to RPL13a. (b) Representative western blot analysis of total and phosphorylated A20 (P-A20) 24 hr post-poly (I:C) (1 *μ*g/mL) exposure. (c) Densitometry analysis of total A20 and (d) the ratio of phosphorylated A20 to total A20. 15 *μ*g were loaded per sample and normalized to *β*-actin. Statistical analysis was performed using two-way ANOVA followed by Bonferroni's post hoc test for (a) with (∗) or NS, representing comparison of NT poly (I:C) and Si poly (I:C). Student's *t*-test was used for analysis in (c) and (d). Data is presented as mean + SEM from two independent experiments with conditions in each set in triplicate. Asterisk (∗) represents statistically significant difference (^∗^*p* value of ≤0.05; NS: nonsignificant).

A20 transcription is positively regulated by NF-*κ*B [50, 51]. At the protein level, A20 facilitates some of the inflammatory signal-suppressing properties of TNIP1 though both can act independently in this regard [11]. Given this, we examined A20 expression being especially interested in its levels in TNIP1-deficient, poly (I:C) exposed cells where induction of several NF-*κ*B-regulated genes were upregulated and cell viability was reduced. TNIP1 knockdown alone had no detectable effect on A20 mRNA levels (Figure 5(a)). However, TNIP1 protein deficiency sensitized cells to low-concentration poly (I:C), causing an almost tripling of A20 mRNA levels compared to poly (I:C) exposure alone at 6 hr after its addition. With continued exposure to poly (I:C), control siRNA-transfected cells had increased A20 levels on par with the TNIP1-deficient, poly (I:C)-treated cells. Thus, we examined its levels at 24 hr post-poly (I:C) addition, a time point by which the increase of A20 mRNA in TNIP1-deficient cells had resolved to that of control-transfected cells (Figure 5(a)). Despite earlier higher (6 hr) and then later similar (12 hr, 24 hr) A20 mRNA levels, poly (I:C)-exposed, TNIP1-deficient cells had similar levels of A20 protein. However, there was a significant reduction (~50%) of phosphorylated A20 potentially limiting its anti-inflammatory function (Figures 5(b)–5(d)).

### 3.6. Transcript Increases Downstream of TLR3 Stimulation in TNIP1-Deficient Cells Are in Part Regulated by JNK and p38

**Figure 6:**
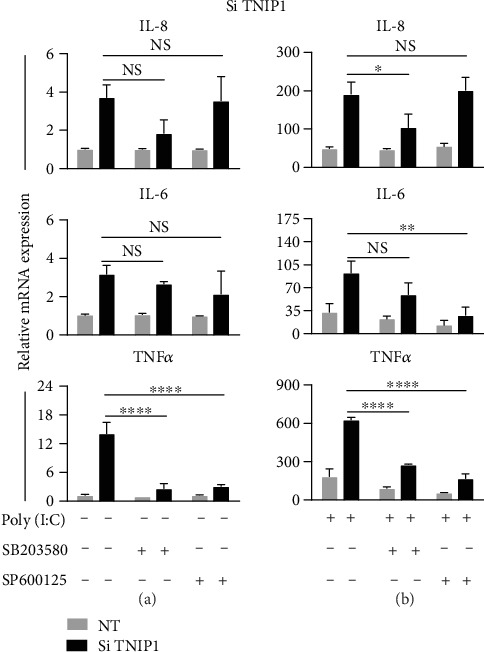
MAPK contributes to increased cytokine gene expression in TNIP1-deficient keratinocytes. Gene expression analysis of HaCaT keratinocytes transfected with TNIP1 siRNA (Si) or nontargeting siRNA (NT) after 6 hr exposure to (a) vehicle or (b) poly (I:C). Two hours prior to poly (I:C) treatment, cells were treated with either JNK (SP600125, 30 *μ*M), p38 (SB203580, 10 *μ*M) inhibitor, or DMSO as vehicle. Data is presented as mean + SEM from two independent experiments with conditions in each set in triplicate. Statistical analysis was performed using two-way ANOVA followed by Bonferroni's post hoc test. Asterisk (∗) represents statistically significant difference (^∗^*p* value of ≤0.05; ^∗∗^*p* value of ≤0.005; ^∗∗∗∗^*p* value of ≤0.0001. NS: nonsignificant).

Keratinocyte activation by agonism of membrane receptors such as TLR3 is induced via both canonical (i.e., NF-*κ*B) and noncanonical (i.e., JNK and p38) signaling pathways [52–54]. TNIP1 deficiency alone (Figure 6(a)) led to modest increases over constitutive levels for IL-8 and IL-6 transcripts but an approximate 12-fold increase for TNF*α*. It was only induction of this latter transcript that showed mediation by p38 and JNK pathways, i.e., statistically significant reduction upon inclusion of their inhibitors SB203580 or SP600125, respectively.

TNIP1-deficient cells treated with poly (I:C) for six hours (Figure 6(b)) had a ~3.5-fold increase in IL-8 gene transcription over that of poly (I:C) alone. Preincubation with a p38 inhibitor significantly reduced that TNIP1 deficiency-dependent exaggerated response, while JNK inhibition had no effect. We found that p38 inhibition had minimal effects on IL-6 gene expression while JNK inhibition promoted diminished expression with a reduction of the significantly induced gene expression found in TNIP1-deficient, poly (I:C)-treated cells. Under conditions of active JNK and p38 pathways, TNIP1 deficiency predisposed keratinocytes to produce an ~4-fold increased TNF*α* gene transcript. p38 or JNK inhibition effectively reduced pathway activity leading to TNF*α* expression due to the combined effect of TNIP1 deficiency and poly (I:C) exposure. Thus, the hyperresponsive expression of these target genes under TNIP1-deficient, TLR3 agonist conditions, appeared to be mostly via p38 for IL-8, JNK for IL-6, and both pathways for TNF*α*.

## 4. Discussion

The surface location of epidermal keratinocytes as well as their own maturation provides for a constant exposure to microbe- and tissue-derived pattern recognition molecules [1]. Despite this constant stimulation, keratinocytes resist being in a chronic responsive state in part by inflammatory signal-suppressing proteins like TNIP1. When overwhelmed by excessive incoming signals (e.g., large scale pathogenic microbe infection and extensive cell-damaging trauma), keratinocytes become activated cells [34–38]. A predicted corollary is that in cases of TNIP1 deficiency, intracellular signal-limiting machinery might be insufficient to halt stimuli from low or “background” levels of DAMPs/PAMPs triggering at least a partially activated state. Activated keratinocytes are rich sources of diverse chemokines, cytokines, antimicrobial peptides, and regulatory factors [35, 46, 55]. In turn, they propagate injury response signals to neighboring epidermal and dermal cells, which if balanced along an appropriate timeline lead to successful tissue recovery but if in excess and/or out of synch may lead to chronic wounds [56, 57] which may lead to pyroptosis [48]. In this report, we present evidence that TNIP1 deficiency synergizes with low levels of poly (I:C), a model of DAMP/PAMP, sensitizing keratinocytes to establish a hyperresponsive inflammatory state compromising reepithelialization and cell viability. TNIP1-deficient HaCaT keratinocytes had altered expression of several keratinocyte-specific genes (TGM1, K1, and K6) in response to poly (I:C) which could be expected to disrupt their performance as protective, terminally differentiating cells or to contribute to effective injury recovery. Additionally, there was significantly increased expression of some genes (e.g., SERPINE, PLAUR, and PLAU) expected to facilitate wound healing but occurring at the same time as increased TNF*α* expression, previously shown to severely reduce keratinocyte viability [58]. There is also coinciding increased gene expression of inflammasome proteins, associated enzymes, and their substrates along with proinflammatory cytokines. Induction of the latter (IL-8, IL-6, and TNF*α*) occurs at least partly through JNK- and p38 MAPK-mediated pathways. In sum, such responses are likely to contribute to a net negative effect on overall wound healing visualized as poor reepithelialization and reduced cell viability.

Some of the most striking degrees of hyperresponsiveness were seen among a cohort of genes involved in overlapping biological states, e.g., EMT, wound healing, and inflammation (Figure 2) especially with factors such as IL-36*γ*, S100A8 and 9, TGF*β*, and CCN2. We found a marked synergy between TNIP1 protein deficiency and poly (I:C) exposure for expression of these. Despite these conditions inducing increased expression of some EMT-associated genes (e.g., TGF*β* and SNAI2), the observed increase in E-cadherin suggests that at best this may be a partial or incomplete EMT as has been associated with wound healing rather than a state of full epithelial-to-mesenchymal transition [35, 59]. Since samples for RNA were collected relatively early (6 hours) after poly (I:C) addition, we recognize that for some of these markers, there may have been second generation signaling events beginning to add to their expression. For instance, CCN2 expression is increased in response to injury [43] as would be simulated by the model DAMP poly (I:C) as well as keratinocyte exposure to TGF*β* [43]. CCN2 can upregulate mRNA for integrins *α*5 and *β*1 [60], two transcripts we saw increased in the wound healing array. Interestingly, CCN2 expression, like S100A9 (Figure 2), is increased in the epidermal layers of systemic sclerosis skin [61], a fibrotic disease state associated with TNIP1 SNPs in patient genomes [62] and TNIP1 protein deficiency [15] in lesional skin.

In addition to their expression changes associated with wound repair [63], the cohort of SERPINE (plasminogen activator inhibitor 1), PLAUR (plasminogen activator, urokinase receptor), and PLAU (plasminogen activator, urokinase) are upregulated in psoriasis compared to amounts beneath the limits of detection or at very low levels in healthy epidermis [64]. As compared to HaCaT keratinocytes with usual levels of TNIP1 protein under poly (I:C) conditions, TNIP1-deficient cells exposed to this DAMP/PAMP had increased expression of each of the following: SERPINE, ~9-fold; PLAUR, ~3-fold; and PLAU, ~2-fold. Thus, it is possible that in addition to its signal-limiting role when present in healthy cells experiencing background microbial burden, TNIP1 reduction, as has been reported for psoriatic keratinocytes [12], may compound the inflammatory state of lesions exposed to even low levels of microbes or damaged cell debris. Such cells may be prone to an increasing inflammatory spiral, as we have previously reported that TNIP1 protein half-life is reduced under inflammatory conditions [65]. It is particularly interesting then that these TNIP1-deficient, low DAMP/PAMP conditions yielded decreased phosphorylated A20, a posttranslational modification that increases A20 functionality and recruitment to suppress inflammatory signaling [51, 66]. Thus, as supported by recent findings [11, 67], it will be important to consider individual and pairwise anti-inflammatory roles of TNIP1 and A20 and the possible differences in cell death due to their deficiency or dysfunction [68].

Inflammasomes are multiprotein processing complexes of NLRs or the related AIM2, ASC, and typically caspase 1 which when assembled lead to cleavage of pro- to mature forms of interleukins such as IL-1*β* and IL-18 [48]. Cytokine release is facilitated by pore-forming gasdermin D which also contributes to overall cell degradation [48]. Interestingly, a previous report [49] linked keratinocyte TLR3 agonism by 25 *μ*g/mL poly (I:C) for 24 hr to an ~140-fold induction of IL-36*γ* mRNA compared to vehicle control along with subsequent cell death by pyroptosis. Notably, in a separate study [69], lower concentrations (1.5 *μ*g/mL) could induce IL-36*γ* protein synthesis and release. However, extracellular accumulation to detectable levels required 72-96 hr and there was no appreciable cell death as indicated by unchanged LDH release. In our study, TNIP1-deficient HaCaT keratinocytes exposed to poly (I:C) in a time- and dose-limited (6 hr; 1 *μ*g/mL) manner showed increased induction not only of IL-36*γ* and IL-18 but also NLRP1, NLRP10, ASC, AIM2, caspase, and gasdermin D. Consistent with NLRP1 basal expression and the indispensable role in inflammasome formation in activated keratinocytes [70], we found it expressed under all conditions but further induced by the dual status of TNIP1 deficiency and poly (I:C) exposure. Although further investigation is needed into protein consequences, these gene expression events suggest that poor reepithelization of TNIP1-deficient, TLR3-stimulated HaCaT keratinocytes may stem from inflammasome assembly and pyroptosis. This interpretation is also consistent with TNIP1-resisting necroptotic signaling which in part overlaps with pyroptosis [66]. Keratinocytes express basal levels [70, 71] of inflammasome-related proteins (e.g., NLRP1, procaspase, pro-IL-1*β*, and pro-IL-18) showing that expression of these components is not absolutely dependent on de novo priming by TLR agonists. Despite a general view of TLR3 agonism by poly (I:C) being favorable to wound healing [72], as shown in this report, TNIP1 deficiency appears to have established a state of overreaction to any incoming TLR-mediated signal leading to previously unrecognized negative consequences from excessive or imbalanced responses. While the induction of ASC expression in TNIP1-deficient HaCaT cells could lead to this end, the potential role of products from other induced genes, e.g., NLRP10, should also be considered. NLRP10 has apparent anti- [73, 74] or proinflammatory [75, 76] effects in regulating inflammasome activity, possibly specific to different cell types and/or availability of its partnering proteins. The latter situation is particularly interesting given NLRP10-TNIP1 protein interaction [77] leading to reduction of TNIP1 protein levels. This, compounded by external inflammatory signals instigating degradation of TNIP1 [65] could lead cells on an inescapable negative spiral to cell death.

## 5. Conclusion

TNIP1 deficiency concurrent with low-concentration challenge of a TLR3 agonist led to imbalanced and/or excessive expression of wound healing and proinflammatory markers with reduced cell viability and further cell loss in scratch-wounded populations. We conclude that TNIP1 sufficiency helps establish threshold (resistable) levels for DAMP/PAMP exposure protecting cells from otherwise chronic inflammatory signaling from even low-level exposure to diverse environmental (cell debris, microbe) cues. TNIP1 deficiency or dysfunction could exacerbate intracellular inflammatory signaling to the detriment of wound recovery. In turn, this may initiate or intensify gene expression consequences that could limit tolerance to encounters with cutaneous microbes or recovery from minor damage events.

## Figures and Tables

**Table 1:** TNIP1 deficiency in HaCaT keratinocytes promotes altered expression of genes associated with wound healing. qRT-PCR array results from HaCaT keratinocytes treated with TLR3 agonist poly (I:C) for 6 hr. Transcript fold changes, with values normalized against housekeeping gene transcript RPLP0, were calculated between poly (I:C)-treated HaCaT keratinocytes previously transfected with either nontargeting or TNIP1 siRNA. PA: plasminogen activator; ECM: extracellular matrix; C-X-C: Cys-X-Cys amino acid motif.

Gene symbol	Fold change	Full name; encoded function
SERPINE1	9.38	Serine protease inhibitor clade E member 1, alias PA inhibitor 1 (PAI-1); ECM remodeling
TNFA	9.13	Tumor necrosis factor; proinflammatory cytokine
CXCL11	5.36	C-X-C motif chemokine ligand 11; chemotactic promigratory/inflammatory cytokine
PLAUR	3.29	Urokinase PA receptor; signals for ECM degradation during remodeling
F3	2.46	Coagulation factor III, alias tissue factor; promotes cell migration during remodeling
IL-10	2.45	Interleukin 10; anti-inflammatory cytokine
TGF*α*	2.20	Transforming growth factor alpha; mitogenic polypeptide
ITGA5	2.04	Integrin alpha 5; cell adhesion molecule
IL-6	2.01	Interleukin 6; inflammatory cytokine
PLAU	1.99	Urokinase PA; converts plasminogen to plasmin during ECM remodeling
CXCL5	-2.06	C-X-C motif chemokine ligand 5; chemotactic promigratory factor
CCL2	-3.08	C-C motif chemokine ligand 2; chemotactic cytokine
CXCL2	-3.18	C-X-C motif chemokine ligand 2; chemotactic promigratory factor
F13A1	-3.97	Coagulation factor XIII A chain; crosslinking of clot-forming fibrin
CTSK	-7.06	Cathepsin K; basement membrane and ECM collagenase
MMP7	-8.67	Matrix metallopeptidase 7 alias matrilysin; gelatinase

## Data Availability

All data used to support the findings of this study are included within the article.

## Abbreviations

DAMP: Damage-associated molecular pattern
PAMP: Pathogen-associated molecular pattern
TNIP1: Tumor necrosis factor alpha-induced protein 3, interacting protein 1
NF-*κ*B: Nuclear factor kappa-light-chain enhancer of activated B cells
TLR: Toll-like receptor
Poly (I:C): Polyinosinic:polycytidylic acid
EMT: Epithelial-mesenchymal transition
MMP: Matrix metallopeptidase
EGF: Epidermal growth factor
TGF: Transforming growth factor
NLRP: Nucleotide-binding oligomerization domain-like receptor proteins
A20: Tumor necrosis factor alpha-induced protein 3 (TNFAIP3).
TNF: Tumor necrosis factor.
JNK: C-Jun N-terminal kinase
p38: p38 mitogen-activated protein kinase

## Supplementary Materials

Supplementary Materials Supplementary Table 1: qRT-PCR primers. Supplementary Figure 1: (a) transfection and treatment timeline. (b) Representative western blot analysis of TNIP1 protein levels 48 and 72 hours posttransfection with nontargeting siRNA control (NT) or TNIP1 siRNA (Si). (c) Densitometry analysis of western blots with NT control set to 1 after normalization to *β*-actin. Supplementary Figure 2: relative abundance of keratinocyte-associated genes in HaCaTs. mRNA expression was normalized to RPL13a from normal HaCaT keratinocytes 72 hr after plating at ~40% confluence. Cells were serum rested for the 36 hr immediately prior to collection. Supplementary Figure 3: TNIP1 deficiency in HaCaT keratinocytes promotes altered expression of genes associated with keratinocyte wound healing. (a) Heat map for qRT-PCR array results from HaCaT keratinocytes treated with TLR3 agonist poly (I:C) for 6 hr. Fold change was calculated comparing TNIP1 siRNA-treated cells versus control, normalized against RPLP0. (b) Genes among the highest fold changes across the array. Supplementary Figure 4: TNIP1 deficiency causes loss of cell confluence after 24-hour exposure to poly (I:C). (a) Representative images of initially 100% confluent TNIP1-sufficient (NT) or TNIP1-deficient (Si) HaCaT keratinocytes 24 hr post-poly (I:C) exposure. (b) Quantitation of percentage confluence performed using PHANTAST plugin for ImageJ (FIJI client).
