# Supplementary material for: Enhanced Wound Healing- and Inflammasome-Associated Gene Expression in TNFAIP3-Interacting Protein 1- (TNIP1-) Deficient HaCaT Keratinocytes Parallels Reduced Reepithelialization
Source: Mediators Inflamm. 2020 Apr 21;2020:5919150. doi: 10.1155/2020/5919150 (PMC7191359; doi:10.1155/2020/5919150)
Supplement: Supplementary Materials — Supplementary Table 1: qRT-PCR primers. Supplementary Figure 1: (a) transfection and treatment timeline. (b) Representative western blot analysis of TNIP1 protein levels 48 and 72 hours posttransfection with nontargeting siRNA control (NT) or TNIP1 siRNA (Si). (c) Densitometry analysis of western blots with NT control set to 1 after normalization to β-actin. Supplementary Figure 2: relative abundance of keratinocyte-associated genes in HaCaTs. mRNA expression was normalized to RPL13a from normal HaCaT keratinocytes 72 hr after plating at ~40% confluence. Cells were serum rested for the 36 hr immediately prior to collection. Supplementary Figure 3: TNIP1 deficiency in HaCaT keratinocytes promotes altered expression of genes associated with keratinocyte wound healing. (a) Heat map for qRT-PCR array results from HaCaT keratinocytes treated with TLR3 agonist poly (I:C) for 6 hr. Fold change was calculated comparing TNIP1 siRNA-treated cells versus control, normalized against RPLP0. (b) Genes among the highest fold changes across the array. Supplementary Figure 4: TNIP1 deficiency causes loss of cell confluence after 24-hour exposure to poly (I:C). (a) Representative images of initially 100% confluent TNIP1-sufficient (NT) or TNIP1-deficient (Si) HaCaT keratinocytes 24 hr post-poly (I:C) exposure. (b) Quantitation of percentage confluence performed using PHANTAST plugin for ImageJ (FIJI client). [file 5919150.f1.pdf]

Target	Forward Primer (5' – 3')<br>Reverse Primer (5' – 3')
TNIP1	TCC AGG AGC CCT AGG AGT G<br>AGG GTA GCT CAG CCC CTG
RPL13a	CTA TGA CCA ATA GGA AGA GCA ACC<br>GCA GAG TAT ATG ACC AGG TGG AA
FILAGGRIN	CTG GAC ACT CAG GTT CCC AT<br>TTT CGT GTT TGT CTG CTT GC
TGM1	CCC TCA CCA ATG TCG TCT TC<br>TCA CTG TTT CAT TGC CTC CA
KRT1	GTC TCG AGA AAG GGA GCA AA<br>GCT CCC ATT TTG TTT GCA GT
KRT10	GAG CCT CGT GAC TAC AGC AA<br>ATC TGA AGC AGG ATG TTG GC
KRT5	GGA GCT CAT GAA CAC CAA GC<br>CTG GTC CAA CTC CTT CTC CA
KRT14	GGC CTG CTG AGA TCA AAG AC<br>TCT GCA GAA GGA CAT TGG C
KRT6A	CTG AGA TCG ACC ACG TCA AG<br>CAG CTT GTT CTT GGC ATC CT
KRT6B/C	TGA GCA GAT CAA GAC CCT CA<br>CCA CTT GGT GTC CAG AAC CT
KRT16	GGC CCA GTG AGA TCA AAG AC<br>CAA TCT GCA AAA TGG GCT G
ITGA3	CCT CTT CGG CTA CTC GGT C<br>GGT TGG TGT AGC CAT CGG
INVOLUCRIN	TCT GCC TCA GCC TTA CTG TG<br>ACT GAG GGC AGG GGA GAG
LORICRIN	ACC TGG CCG TCC AAA TAG AT<br>CAA ACC TCG GGT AGC ATC AT
CXCR1	GGC CGG TGC TTC AGT TAG AT<br>GGG CAT AGG CGA TGA TCA CA
IL-20	ATG CCA TTG TGG GGA GGA AG<br>CCC AAA GCC TTC ACA ACT GC
S100A8	ATG CCG TCT ACA GGG ATG AC<br>ACG CCC ATC TTT ATC ACC AG
S100A9	GGA ATT CAA AGA GCT GGT GCG<br>AGC TGC TTG TCT GCA TTT GTG
CXCR1	TAC TGT TGG ACA CAC CTG GC<br>ATC CCA CAT CTG TGG ATC TGT

Target	Forward Primer (5' – 3')<br>Reverse Primer (5' – 3')
TGFβ1	CCC TGG ACA CCA ACT ATT GC<br>CTT CCA GCC GAG GTC CTT
SNAI2	GAG CAT ACA GCC CCA TCA CT<br>GGG TCT GAA ACT TGG ACT G
CCN2	GAC CTG GAA GAG AAC ATT AAG AAG G<br>TCG GTA TGT CTT CAT GCT GGT G
PERIOSTIN	CAG TTT TGC CCA TTG ACC ATG<br>ATA GCG CTG CGT TGT GGT G
E-CADHERIN	TTC TCC GCC TCC TTC TTC AT<br>TTC TGC TGC TCT TGC TGT TT
NLRP1	CAG GCA GCA CAG ATC AAC AT<br>GTG ACC TTG AGG ACG GAG AA
AIM2	CAG AAA TGA TGT CGC AAA GCA<br>TCA GTA CCA TAA CTG GCA AAC AG
CASPASE 1	GCC TGT TCC TGT GAT GTG GAG<br>TGC CCA CAG ACA TTC ATA CAG TTT C
IL-36γ	GGG CCG TCT ATC AAT CAA TG<br>TGA TAA CAG CAA CAG TGA CTG
IL-18	CAA TTG CAT CAA CTT TGT GGC<br>TAA ATA TGG TCC GGG GTG CA
NLRP10	GGA GGC TGT GAA AGT TGT CC<br>ATC TGC CAT TGA CTC CTG CT
ASC	AGT TTC ACA CCA GCC TGG AA<br>TTT TCA AGC TGG CTT TTC GT
GASDERMIN D	GAG TGT GGC CTA GAG CTG G<br>GGC TCA GTC CTG ATA GCA GTG
IL-1β	GAA GCT GAT GGC CCT AAA CA<br>AAG CCC TTG CTG TAG TGG TG
A20	GGG GCC CGG AGA GGT AAA<br>GAG GAA GGA CTT GTT CAG CCA
IL-8	GGA GAG TTT TTG AAG AGG GCT GA<br>TGC TTA AGT TTC ACT GGC ATC TT
IL-6	GGT ACA TCC TCG ACG GCA TCT<br>GTG CCT CTT TGC TGC TTT CAC
TNFα	CTG CTG CAC TTT GGA GTG AT<br>AGA TGA TCT GAC TGC CTG GG

Supplementary Table 1. qRT-PCR primers.

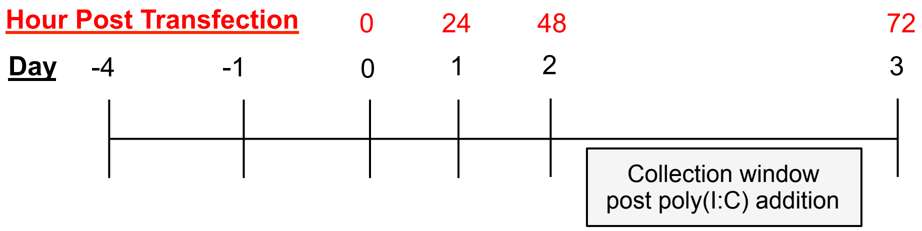

Day	Experimental Action taken
-4	Cells sub-cultured at a 1/20 dilution from 100% confluent plate
-1	Cells plated in 12-well or 24-well plate at final ~40-60% Confluent
0	Cells transfected with either non-targeting or TNIP1 siRNA
1	Serum rest period begins with media change into serum free media
2	Media is removed and replaced with media containing either vehicle control or poly(I:C) at 1 μg/mL
3	Final collection point for cells

(a)

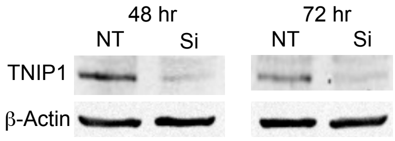

(b)

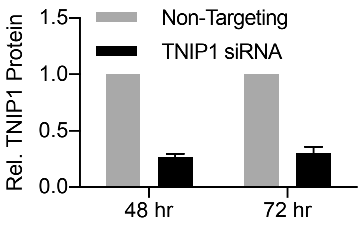

(c)

Supplementary Figure 1. (a) Transfection and treatment timeline. (b) Representative western blot analysis of TNIP1 protein levels 48 and 72 hours post transfection with non-targeting siRNA control (NT) or TNIP1 siRNA (Si). (c) Densitometry analysis of western blots with NT control set to 1 after normalization to β-actin. Data presented as mean + SEM from two independent experiments.

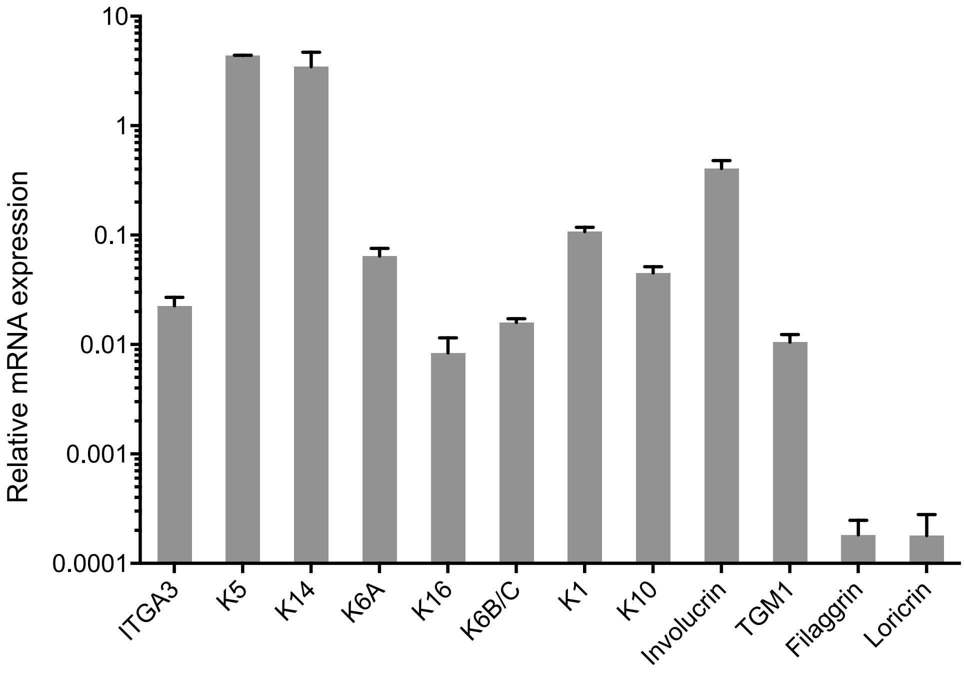

Supplementary Figure 2. Relative abundance of keratinocyte-associated genes in HaCaTs. mRNA expression normalized to RPL13a from normal HaCaT keratinocytes 72 hr after plating at ~40% confluence. Cells were serum rested for the 36 hr immediately prior to collection. Data presented as mean + SEM from two independent experiments with conditions in each set in triplicate.

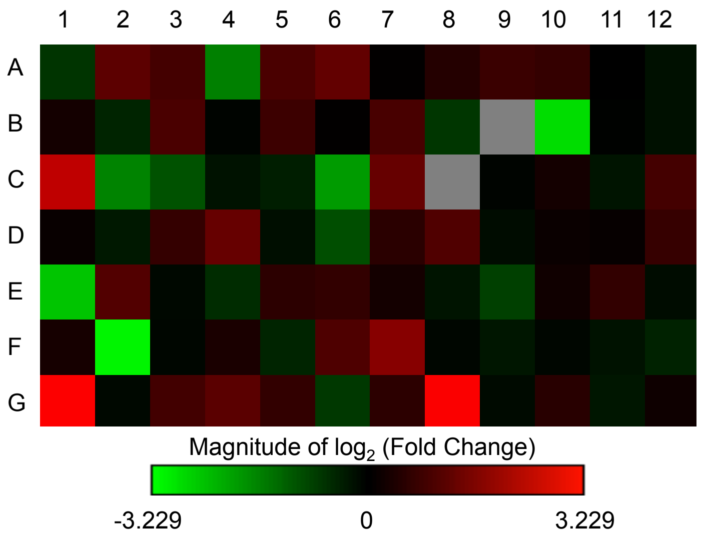

(a)

	01	02	03	04	05	06	07	08	09	10	11	12
A	ACTA2 -1.59	ACTC1 2.24	ANGPT1 1.83	CCL2 -3.08	CCL7 1.92	CD40LG 2.38	CDH1 1.01	COL14A1 1.38	COL1A1 1.66	COL1A2 1.57	COL3A1 -1.00	COL4A1 -1.15
B	COL4A3 1.19	COL5A1 -1.37	COL5A2 1.92	COL5A3 -1.03	CSF3 1.70	CSF3 1.02	CTGF 1.89	CTNNB1 -1.62	CTSG -1.00	CTSK -7.06	CTSV -1.02	CXCL1 -1.15
C	CXCL11 5.36	CXCL2 -3.18	CXCL5 -2.06	EGF -1.17	EGFR -1.29	F13A1 -3.97	F3 2.46	FGA -1.00	FGF10 -1.03	FGF2 1.20	FGF7 -1.19	HBEGF 1.81
D	HGF 1.08	IFNG -1.23	IGF1 1.57	IL10 2.45	IL1B -1.14	IL2 -1.99	IL4 1.43	IL6 2.01	IL6ST -1.11	ITGA1 1.08	ITGA2 1.05	ITGA3 1.62
E	ITGA4 -5.72	ITGA5 2.04	ITGA6 -1.07	ITGAV -1.48	ITGB1 1.47	ITGB3 1.55	ITGB5 1.19	ITGB6 -1.20	MAPK1 -1.76	MAPK3 1.15	MIF 1.56	MMP1 -1.12
F	MMP2 1.21	MMP7 -8.67	MMP9 -1.05	PDFGA 1.26	PLAT -1.36	PLAU 1.99	PLAUR 3.29	PLG -1.06	PTEN -1.21	PTGS2 -1.06	RAC1 -1.17	RHOA -1.34
G	SERPINE1 9.38	STAT3 -1.08	TAGLN 1.79	TGFA 2.20	TGFB1 1.54	TGFBR3 -1.64	TIMP1 1.47	TNF 9.13	VEGFA -1.10	VTN 1.42	WISP1 -1.20	WNT5A 1.13

(b)

Supplementary Figure 3. TNIP1 deficiency in HaCaT keratinocytes promotes altered expression of genes associated with keratinocyte wound healing. (a) Heat map for qRT-PCR array results from HaCaT keratinocytes treated with TLR3 agonist poly (I:C) for 6 hr. Fold change calculated comparing TNIP1 siRNA treated cells versus control, normalized against RPLP0. Grey boxes: amplicons with a Ct value greater than set maximum (>35 Ct). (b) Genes among the highest fold changes across array.

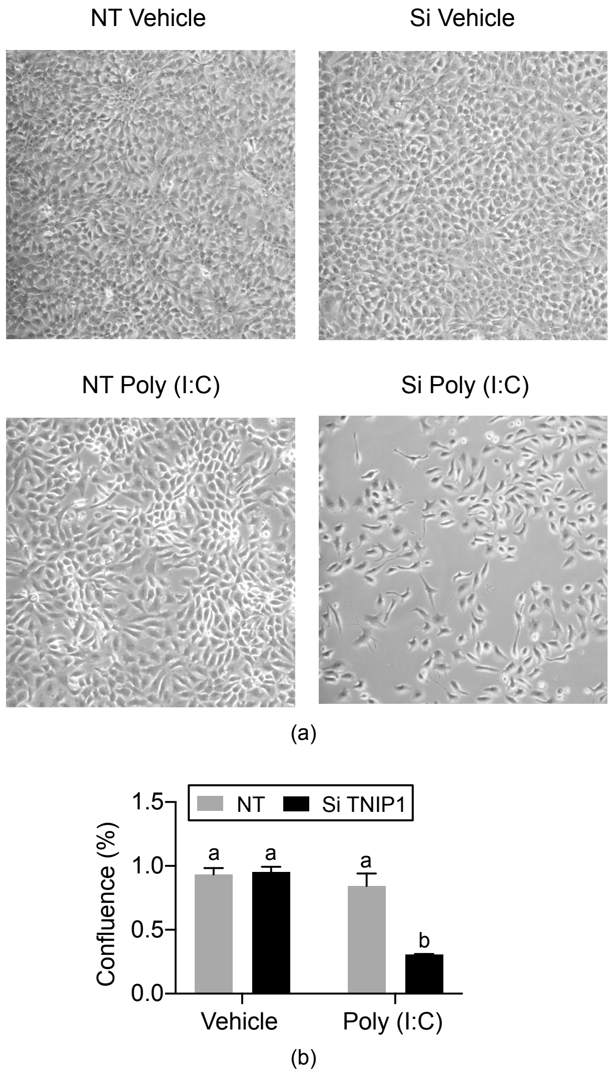

Supplementary Figure 4. TNIP1 deficiency causes loss of cell confluence after 24 hour exposure to poly (I:C). (a) Representative images of initially 100% confluent TNIP1 sufficient (NT) or TNIP1 deficient (Si) HaCaT keratinocytes 24 hr post-poly (I:C) exposure. (b) Quantitation of percentage confluence performed using PHANTAST plugin for ImageJ (FIJI client). Total of N=15 images were analyzed for each condition taken from two independent experiments with conditions in each set in triplicate. Means with a common letter are not significantly different by two-way ANOVA followed by Tukey's post hoc-test at a significance level of $p \leq 0.05$.
